# Supplementary material for: High-resolution mapping of fluoroquinolones in TB rabbit lesions reveals specific distribution in immune cell types
Source: eLife. 2018 Nov 14;7:e41115. doi: 10.7554/eLife.41115 (PMC6249001; doi:10.7554/eLife.41115)
Supplement: Figure 4—source data 1. [file elife-41115-fig4-data1.docx]

$PROBLEM Final model

$INPUT ID HIS LYM NEC NEU EPI RAT DISI DISV DV MED SD

;ID = Lesion ID

;HIS= histiocytes (also referred to as macrophages throughout the manuscript)

;LYM= lymphocytes

;NEC= necrotic cells

;NEU= neutrophils

;EPI= epithelial cells

;RAT= distance ratio or ratio between the distance from ROI to outer granuloma edge and distance from outer caseum border to granuloma edge

;DISI= absolute distance from ROI to granuloma edge in pixels

;DISV= absolute distance from ROI to granuloma edge in μm

;MED = average pixel intensity normalized to internal standard (or drug abundance)

;SD = standard deviation of the abundance

$DATA moxi.data.04082018.nm.csv IGNORE=@ IGN(DV.EQ.-99)

$PRED

;;; S0HIS-DEFINITION START

S0HIS = EXP(THETA(7)*(HIS - 0.35))

;;; S0HIS-DEFINITION END

;;; S0RAT-DEFINITION START

S0RAT = EXP(THETA(6)*(RAT - 0.65))

;;; S0RAT-DEFINITION END

;;; S0NEC-DEFINITION START

IF(NEC.EQ.0) S0NEC = 1 ; Most common

IF(NEC.EQ.0.05) S0NEC =(1+THETA(3))

IF(NEC.EQ.0.3) S0NEC = ( 1 + THETA(4))

IF(NEC.EQ.0.5) S0NEC = ( 1 + THETA(4))

IF(NEC.EQ.0.75) S0NEC = ( 1 + THETA(4))

IF(NEC.EQ.0.8) S0NEC = ( 1 + THETA(4))

IF(NEC.EQ.0.9) S0NEC = ( 1 + THETA(5))

IF(NEC.EQ.1) S0NEC = ( 1 + THETA(5))

;;; S0NEC-DEFINITION END

;;; S0-RELATION START

S0COV=S0NEC*S0RAT*S0HIS

;;; S0-RELATION END

;###### Base model #######

TVS0 = THETA(1)

TVS0 = S0COV*TVS0

S0 = TVS0*EXP(ETA(1))

IPRED=S0

W=THETA(2)

IRES=DV-IPRED

IWRES=IRES/W

Y = IPRED*(1 +W*EPS(1))

$THETA (0,0.0104602) ; 1 Baseline

$THETA (0,0.494232) ; 2 Residual variability, SD

$THETA (-1,0.0716992,5) ; S0NEC1

(-1,0.10305,5) ; S0NEC2

(-1,0.10305,5) ; S0NEC3

$THETA (-1000000,-0.99504,1000000) ; S0RAT1

$THETA (-1000000,0.824672,1000000) ; S0HIS1

$OMEGA 0.0217007 ; 1 Baseline

$SIGMA 1 FIX ; RV Distirbution

$ESTIMATION METHOD=1 PRINT=1 INTER MAXEVAL=9990

$COV

$TABLE ID DV TVS0 MED SD ETA1 IPRED IWRES HIS LYM NEC NEU EPI RAT DISI DISV NOPRINT ONEHEADER FILE=sdtab14
