# Supplementary material for: High-resolution mapping of fluoroquinolones in TB rabbit lesions reveals specific distribution in immune cell types
Source: eLife. 2018 Nov 14;7:e41115. doi: 10.7554/eLife.41115 (PMC6249001; doi:10.7554/eLife.41115)
Supplement: Supplementary file 1. — (A) Spearman rank correlation analysis of MXF abundance in 35 regions of interest (ROIs, Figure 3—figure supplement 2) versus seven recorded parameters. (B) Final model parameters. (C) Model parameters obtained with training dataset only. [file elife-41115-supp1.docx]

**Supplementary file 1A**. Spearman rank correlation analysis of MXF abundance in 35 regions of interest (ROIs, **Figure Supplement 5**) versus 7 recorded parameters.

|  | MXF  vs % histiocyte | MXF  vs  % lymphocyte | MXF  vs  % necrosis | MXF  vs  % neutrophil | MXF  vs  % epithelial cell | MXF  vs  distance ratio ^(1)^ | MXF  vs absolute distance |
| --- | --- | --- | --- | --- | --- | --- | --- |
| Spearman ρ | 0.8365 | 0.1237 | -0.6643 | -0.05117 | 0.2728 | -0.7196 | -0.6229 |
| 95% C.I. | 0.6924 to 0.9164 | -0.2283 to 0.4471 | -0.8201 to -0.4168 | -0.3867 to 0.2963 | -0.0767 to 0.5626 | -0.852 to -0.5006 | -0.7956 to -0.3566 |
| *p* (two-tailed) of likelihood ratio test | <0.0001 | 0.4791 | <0.0001 | 0.7703 | 0.1129 | <0.0001 | <0.0001 |
| *p* value summary | **** | ns | **** | ns | ns | **** | **** |
| d.f. (n-2) | 33 | 33 | 33 | 33 | 33 | 33 | 33 |

C.I.: confidence interval

^(1)^ relative distance of each ROI to the outer edge of the granuloma, calculated as the ratio between the distance of each ROI to the outer border and the total distance between the inner and outer borders of the cellular rim.

**Supplementary file 1B.** Final model parameters

| Model parameters | Base model (no predictors) | Full model (with predictors) |
| --- | --- | --- |
|  | median (relative standard error (%)) | |
| MXF abundance (Θ_1_) ^(1)^ | 0.01224 (9) | 0.0104 (4) |
| Between-ROI variability | 49% (8) | 18.9 (15) |
| Within-ROI variability | 43.3% (10) | 49.4% (8) |
| Distance Ratio (Θ_2_) ^(1)^ | n.a. | -0.9864 (9) |
| % Histiocytes (Θ_3_) ^(2)^ | n.a. | 0.8886 (12.2) |
| % Necrotic cells (Θ_necrosis_) |  | |
| Θ_necrosis_  if % necrosis  ≤ 0.05 | n.a. | 0.0722 (94) |
| Θ_necrosis_ if % necrosis > 0.05 and < 0.9 | n.a. | 0.2232 (54) |
| Θ_necrosis_  if % necrosis ≥ 0.9 | n.a. | 0.2965 (82) |

**Supplementary file 1C.** Model parameters obtained with training dataset only

| Model parameters | Base model (no predictors) | Full model (with predictors) |
| --- | --- | --- |
|  | median (relative standard error (%)) | |
| MXF abundance (Θ_1_) ^(1)^ | 0.01275 (11) | 0.01023 (6) |
| Between-ROI variability | 44% (13) | 18.8 (20) |
| Within-ROI variability | 47.4% (10) | 47.4% (10) |
| Distance Ratio (Θ_2_) ^(1)^ | n.a. | -1.151 (8) |
| % Histiocytes (Θ_3_) ^(2)^ | n.a. | 0.8645 (13) |
| % Necrotic cells (Θ_necrosis_) |  | |
| Θ_necrosis_  if % necrosis ≤ 0.05 | n.a. | 0.0857 (93) |
| Θ_necrosis_ if % necrosis > 0.05 and < 0.9 | n.a. | 0.2146 (36) |
| Θ_necrosis_  if % necrosis ≥ 0.9 | n.a. | 0.4967 (36) |

n.a.: not applicable

^(1)^ Median of MXF abundance across 35 ROIs; the value shown in the full model column is adjusted to account for the most likely values of the predictors

^(2)^ Ratio between distance of ROI to outer edge of granuloma and total distance from outer edge to caseum

^(3)^ includes macrophages and foamy macrophages
